# Congruence between Physical Activity Patterns and Dietary Patterns Inferred from Analysis of Sex Differences in Lifestyle Behaviors of Late Adolescents from Poland: Cophylogenetic Approach

**DOI:** 10.3390/nu15030608

**Published:** 2023-01-24

**Authors:** Jarosław Domaradzki

**Affiliations:** Department of Biostructure, Wroclaw University of Health and Sport Sciences, 51-612 Wrocław, Poland; jaroslaw.domaradzki@awf.wroc.pl

**Keywords:** late adolescents, physical activity patterns, dietary patterns, healthy and unhealthy behaviors, sex differences in lifestyle

## Abstract

Global trends toward physical inactivity and the replacement of healthy dietary behaviors with unhealthy food consumption, particularly in late adolescence, were commonly observed. Thus, the aim of this work was twofold: (1) to examine congruence between PAPs and DPs by identifying sex differences in healthy and unhealthy behaviors and (2) to assess the explanation behind why certain participants were classified into clusters using BMI and fat mass covariates. Late adolescents (19–21 years of age, *n* = 107) were selected to study. PAPs and DPs were assessed with questionnaires (IPAQ, QEB) and body height, weight, and fat mass percentage were self-reported (the accuracy and reliability of self-reported data were empirically verified). A cophylogenetic approach with several clustering procedures (heatmaps and tanglegrams) was the way to solve the stated problems. Results showed that students achieved the minimal level of physical activity, lower than students from other regions in Poland, Czech Republic, or Germany. There was congruence between PAPs and DPs in some males’ and females’ behaviors. Significant similarities in sex patterns of behaviors were revealed (Mantel tests–PAPs: r = 0.70, *p* < 0.001; DPs: r = 0.84, *p* < 0.001). Similarities in PAPs were related to transportation cycling and school/work activities behaviors. Non-healthy triads in dietary behaviors were found: fast-food, sweetened beverages, and alcoholic drinks in the first, and sweets, fried meals, and yellow cheese in the second. One healthy diad was revealed: vegetables and fruits. Only females’ dietary behaviors were reflected in body mass index (F = 3.19, *p* = 0.049), percentage of body fat (F = 3.87, *p* = 0.027), and fat mass index (F = 3.83, *p* = 0.028). The usefulness of the cophenetic approach in patterns study was verified. Sex similarities and specific disparities could help preparing targeted tailored intervention programs that improve healthy habits in late adolescents. This is especially important in relation to females, who more strongly reflected dietary behaviors in body composition.

## 1. Introduction

There is growing interest in the study of the relationship between physical activity (PA) and dietary behaviors and their combined effect on body weight, body composition, and various health markers [1,2,3]. Evidence from scientific studies clearly shows that the number of overweight and obese boys and girls aged 5–19 years has increased by approximately tenfold from 1975 to 2016 [4]. Maintaining an appropriate body weight (in proportion to body height) is important, particularly in adolescence, which is usually recognized as a period needing special attention, as it is one of the most rapid phases of human development [5]. Because somatic and physiological maturity precedes mental maturity reflecting social behavior in adulthood, this sensitive phase of development can make adolescents particularly vulnerable when their capacities are still developing and they are beginning to move outside the confines of their families. Vulnerability to external stressors reflects endocrinal transformations, which results in greater biological and mental susceptibility to factors of the external environment (physical and social) [6]. Besides the obvious relationship between weight and health, a good weight-to-height proportion improves the self-esteem of young people [7,8].

PA is defined as any bodily movement produced by the skeletal muscles that result in energy expenditure [9]. PA has been found to have comprehensive health benefits for people of all ages. Evidence has shown that teenagers and young adults leading 60 min of moderate-to-high-intensity PA each day gain significant health benefits in physical capacity, motor performance, and body mass composition [10]. However, physical inactivity is increasing among adolescents and causing an increase in the prevalence of individuals that are overweight or obese [11]. Excess weight was proved as a risk factor of cardiovascular disease, type 2 diabetes and musculoskeletal disorders [12]. The worldwide phenomenon of the still-growing number of obese children and adolescents has also been observed in Poland, where regular PA has declined to a greater degree than that seen in other European and world countries. Being physically active is a critical habit necessary to be healthy. Numerous studies have documented the many benefits of a physically active lifestyle on health and well-being [13,14].

A balanced diet is commonly perceived as a second, next to PA, component of lifestyle and a contributing factor of health, defined as the long-term average of nutrients and food consumed in a day. A poor diet related to poor choices in food consumption or the lack of access to healthy foods is the main reason for weight problems and health disorders. Being overweight, obese, or underweight is the primary cause of the deterioration of global health. Overnutrition and malnutrition (particularly in developing nations) are widespread across the globe and affect individuals in every country and region of the world [15]. Scientific evidence suggests that the global trend of a steadily increasing percentage of both overweight and underweight individuals is increasingly affecting children and young people [4,16]. One of the main contributors to overweight and obesity in late adolescents is the replacement of a healthy diet (rich in fish, fruits, and vegetables) with energy-dense foods that are high in fat and sugar. Although it is common for young people, the phenomenon was particularly intense among students beginning college [17]. Changes in DPs include an increase in food intake with an increase in the use of edible oils and sugar-sweetened beverages in college students [18].

The multidimensional characteristics of a daily diet require a comprehensive assessment of food consumption. The most common way to evaluate the most frequently consumed foods is by studying DPs [19,20]. The same conclusion can be drawn from PA studies. PA and dietary behaviors have been found to have a parallel relationship during adolescence [2]. Active people tend to have healthier diets [21]. Therefore, studying PA and dietary behaviors together can give a more in-depth insight into the lifestyles of late adolescents.

The relationship between PA and dietary behaviors has been studied many times [2,21,22,23,24]. Patterns of PA (or inactivity) and eating behaviors in young people reveal a mixture of healthy and unhealthy behaviors [25]. It is unknown if the relationship between PA and dietary behaviors has a synergistic and additive effect on body weight/body composition. Clustering individuals (based on variables characterizing PA and dietary intake) allows the identification of people with similar physical activity and dietary patterns. In the identified clusters, it is crucial to determine the underlying mechanisms in the alterations of PA and dietary patterns. Inadequate PA and/or inappropriate diet can lead to disturbances in body composition and, in effect, in health.

To the best of the author’s knowledge, there are only a few studies related to PA and dietary patterns comparisons in late adolescence (young adults aged 18–21+ [8]) in the Polish population, specifically in the population of students who recently started studying. This situation oftentimes disrupts the regular rhythm of the day and can cause abnormalities in eating patterns. There are no studies concerning the congruence between PA and dietary patterns as well as sex disparities in lifestyle behaviors in physically active populations defined by fields of study such as sports or physical education. These students are guaranteed a certain amount of PA that is reflected in job-related MET calculations. Moreover, there are a lack of studies presenting relationships and correspondence between patterns of PA and dietary behaviors at the same time by clustering these individuals based on patterns. Recent approaches have been based on principal component analyses (PCA) [26,27] or logistic regression [2]. Another valuable solution is using two-way clustering and presenting tanglegrams between the two patterns. Such an approach has not been done yet. This cophenetic approach is useful because of the easy visual assessment of the relationship between two trees. In addition, several cophenetic statistics support the decision related to the hypothesis. Only a few studies implemented, e.g., cophenetic correlations to assess the stability of cluster solutions [24].

This study focused on assessing the patterns of PA and DI in male and female students, as well as studying the problem of whether body mass composition reflected PA and dietary behaviors. Therefore, the aim of this study was twofold: (1) to examine congruence between PAPs and DPs, identifying sex differences in healthy and unhealthy behaviors, and (2) to assess the explanation behind why certain participants were classified into clusters using BMI and fat mass covariates. Specifically, detailed questions were used: 1. What are the PA and dietary behaviors in late adolescents who started university study in PE- and sport-oriented fields? 2. Is there congruence (similarity) in PAPs and DPs between males and females? 3. Are the PAPs and DPs reflected in body mass composition?

It was hypothesized that there is congruence between particular PAPs and dietary behavior patterns and that body mass composition reflects lifestyle.

## 2. Materials and Methods

### 2.1. Sample Size and Power Calculation

The main statistical methods used in this article were cluster analyses. While there is no suitable power analysis for clustering methods, the rule recommended by Dalmaijer et al. [28] was taken into account. It is recommended to have 15–30 participants per anticipated subgroup. Hypothetically, four clusters were expected in the main analysis, in each sex. Thus, a minimum of 60 males and 60 females should be examined. Finally, 107 participants were examined (see participants) and, therefore, cluster analysis assumption was slightly violated, which was included in the limitations of this work.

### 2.2. Ethics

This study was approved by the Research Bioethics Committee of the Faculty Senate at the Wroclaw University of Health and Sport Sciences (consent numbers 33/2018 and 13/2022). It was conducted in accordance with the ethical principles for medical research involving human subjects contained in the Declaration of Helsinki published by the World Medical Association. The study also met the “Ethical standards in sport and exercise science research” [29]. All participants were asked to provide informed consent (in an online form) prior to the study and the purpose and characteristics of the research were explained.

### 2.3. Study Design

The research was carried out at the beginning of 2022 in the Biokinetics Research Laboratory (part of the Central Research Laboratory) of Wroclaw University of Health and Sport Sciences. This facility has Quality Management System Certificates PN-EN ISO 9001: 2009 (Certificate Reg. No.: PW-48606-10E) and PN-EN ISO 9001:2015 (Certificate Reg. No.: PW-15105-22X). A non-probability sampling technique was used to acquire students. Participants included in the study passed a three-step procedure: the first step—was consent to participate in the study, the second step—was to complete the online survey, and in the third step—anthropometric and body composition measurements were conducted for a selected fraction of participants. It was not possible to perform the measurements on all of the students, but it was key to evaluate the accuracy of the self-reported data (particularly BFP). Therefore, a representative subset of randomly chosen participants was used to assess the convergence between self-reported and empirically measured data. The author of this study prepared the online questionnaires and conducted anthropometric and body composition measurements.

### 2.4. Participants

**Figure 1:**
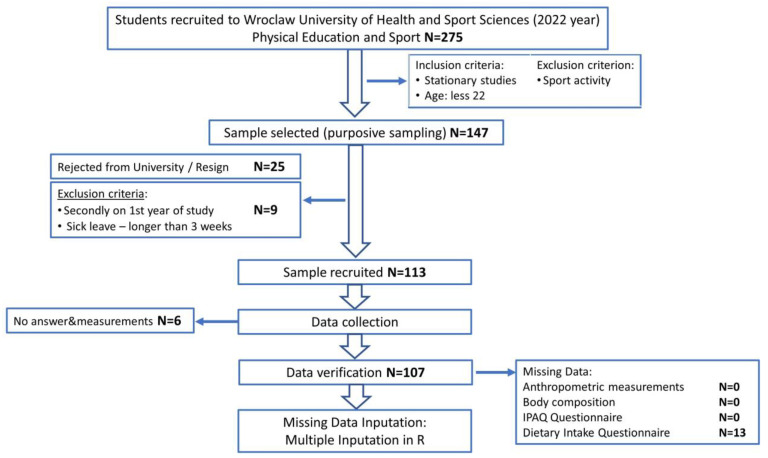
Flowchart: study design and data collection.

Participants included 107 healthy individuals, of which 52 were males (48.6%). Students in their 1st year of study in the Faculty of Physical Education and Sport at Wroclaw University of Health and Sport Sciences were recruited in 2022. Although the proportion did not represent the true population in this field of study (there is a slight bias towards females), a posteriori data-driven, exploratory approach justified leaving all acquired males in the analysis. Students were recruited during the 2021/2022 academic year. Participants interested in the study received a personal link to the questionnaires and were included in the anthropometric and body composition measurements. A flowchart (Figure 1) presents the full sampling procedure.

Preliminary inclusion criteria included students attending classroom courses who were less than 22 years of age. The exclusion criterion included those that participated in a regulated sporting activity through the university and athletes with sport-classes or a mastery level class. A total of 147 students were accepted to take part in the examinations. The subgroup that was rejected by the university (*n* = 25) was identified and removed from the list of participants. To increase the homogeneity of the group, additional exclusion criteria were established, which included remediation of the first year of study and a sick leave longer than 3 weeks from the beginning of the examination. This resulted in an additional 9 students being excluded from the list, resulting in a total sample of 113 participants. Despite providing interest in joining the research, 6 students did not respond to the questionnaires or did not take part in anthropometrical measurements. Therefore, data from 107 participants were collected.

### 2.5. Data Collection

Data were collected using the Student Health Behaviors Studies (STUHB22) local project which evaluates PA, attitudes toward health, lifestyle, and intrinsic and extrinsic risk factors for injuries during PA, dietary behaviors, and overweight and obesity risk factors. Closed-question questionnaires were used. The study was conducted on students of sport field studies using online Google forms immediately after an academic lecture (Human Anatomy taught by the author) during the 2022 academic year. Recruitment, data collection, and entry were conducted by the author of this article. During this pilot-study project, a 20% subset of students consisting of 19 individuals—9 men and 10 women—were selected using a simple random selection (using R function (sample) and alphabetical list) procedure and were measured by the author (anthropometrical and body composition measurements). Using the statistical approach, agreement the relationship between empirical and self-reported data (body height, weight, and BFP) was assessed.

### 2.6. Questionnaires Measurements

Questionnaires were prepared on Google forms in accordance with the rules presented for the document. Polish versions of the questionnaires were used.

#### 2.6.1. Physical Activity

The Polish version of the International Physical Activity Questionnaire (IPAQ), long-form, was used [30]. The IPAQ is a validated, well-known, and easy-to-use questionnaire. The questionnaire consisted of 11 items accessing PA that are separated into four domains measuring school or work, transport, housework/gardening, and leisure time activity levels. The twelfth item was time spent sitting. The duration and frequency of PA during the 7 days preceding the examinations were gathered. The information was introduced into a formula to calculate the Metabolic Energy Turnover (MET) in MET-min/week. Constant coefficients of MET values were assigned to each item of PA. According to the IPAQ user manual, a score for each domain and overall total PA (MET-min/week) as a sum of the scores of all four domains were calculated. The scoring protocol enabled the use of categorical variants of the calculations divided into levels of PA: low (<600 MET-min/week), moderate (600–2999 MET-min/week), and high (≥3000 MET-min/week).

#### 2.6.2. Dietary Characteristics

Information on the frequency of consumption of the selected food groups in the one year preceding examinations was received by food frequency method from the self-administrated Questionnaire of Eating and Behaviors (QEB) [31]. Internal reliability of the QEB was assessed as great with Fleiss’ kappa from 0.64 to 0.84 [32]. Although a complete set of questions related to dietary habits consists of selected 21 food groups (21 questions), the minimal set of 16 questions recommended in the instruction of the QEB was used in this work [33]. The frequency of consumption of each food group was expressed using 6 measurement categories: never, 1–3 times per month, once per week, several times per week, daily, and several times per day. Each category was converted to coefficients and the frequency of consumption was expressed as times/day (never = 0, 1–3 times per month = 0.06, once per week = 0.14, several times per week = 0.5, daily = 1, and several times per day = 2). Indexes on diet quality (modules) were calculated into a pro-healthy dietary habits index (8 items: wholegrain bread, milk, fermented milk drinks, curd cheese (including homogenized cheese), fish and fish dishes, bean and pea dishes, fruits, and vegetables) and an unhealthy dietary habits index (fast food, fried foods, cheese (including cream cheese), sweets, confectionery, canned meat, canned fish or canned vegetable-meat, sweetened carbonated beverages, energy drinks, and alcoholic drinks).

### 2.7. Anthropometric and Body Composition Measurements

Participants were asked about their body height, body weight, and BFP. Students were measured during the students’ classes (e.g., anthropometric, anthropology, weightlifting, light athletics, team game sports) as a part of the academic study program. To assess the reliability of self-reported data a fraction of the participants (20%) were measured by the author to compare empirical and self-reported data.

Two body height measurements were taken with an accuracy of 0.1 cm using an anthropometer (GPM Anthropological Instruments). Body weights and body fat percentages were measured with a body composition analyzer using the InBody230 bioelectric impedance method (InBody Co., Ltd., Cerritos, CA, USA).

$$\mathrm{BMI}=\frac{\mathrm{body}\;\mathrm{mass}\;\left[\mathrm{kg}\right]}{{\mathrm{body}\;\mathrm{height}\;[m}^{2}]}$$

$$\mathrm{FMI}=\frac{\mathrm{body}\;\mathrm{fat}\;\mathrm{mass}\;\left[\mathrm{kg}\right]}{{\mathrm{body}\;\mathrm{height}\;[m}^{2}]}$$

### 2.8. Handling and Imputation of Missing Data

Although there were no missing data in the anthropometrical and body composition self-reported and measured dataset, as well as in the PA questionnaire, there were missing data on the QEB (*n* = 13). Since cluster analysis requires there to be no missing data, all measurements were preprocessed by applying multiple imputations. In this study, the propensity for a data point to be missing was completely random, also known as missing completely at random (MCAR) [34,35]. There was no relationship between whether a data point was missing and any other values in the data set.

Imputation was conducted in R language using RStudio software v. 2022.7.1.554 (RStudio Team (2022). RStudio: Integrated Development Environment for R. RStudio, PBC, Boston, MA, USA URL http://www.rstudio.com/ (accessed on 15 November 2022)) with package *mice* (v.3.14.0).

### 2.9. Validation and assessment of the Consistency between Self-Reported and Empirically Measured Body Weight and Percentage of Body Fat

To decide whether self-reported body fat percentages and body weights were trustworthy to be used in the analyses we conducted statistical procedures for testing intercepts and slopes were applied [36]. The basis for the validation procedure in examining the agreement and consistency of the self-reported and empirically measured data was to perform hypothesis testing to determine whether the differences between constants and coefficients using two different regression models (self-reported and empirically measured data) were statistically significant. Two questions that should be answered included (1) whether the two models have different constants (null hypothesis: there is no vertical shift between regression lines) and (2) did the way of registering data (self-reporting and measuring) affect the relationship between body weight and BFP. The assumption for this procedure was that empirically measured data is trustworthy and that the relationship between body weight and body fat is strong and certain. Comparisons of self-reported data allowed for the acceptance or rejection of body fat percentages from the analyses. Regressions conducted for the male and female groups are presented on Figure 7.

### 2.10. Statistics

Statistical characteristics of the male and female groups for continuous variables were presented as means, medians, and 95% confidence intervals (CI). Categorical variables were presented as numbers and percentages. The CI formula for medians came from Conover [37]. To test differences between the two groups of participants (e.g., sex differences), Student t-tests were used for continuous normally distributed data, while the Mann–Whitney U test was used for non-normally distributed data (frequency of food consumption). Relationships between categorical data were tested with *χ*^2^ tests. The Shapiro–Wilk test was used to evaluate the normality of data distribution.

Before conducting analyses, the variables identified as non-normally distributed were transformed to receive a normal shape of the distribution. Transforming data to bring them closer to normality was needed to meet the assumptions for the other statistical methods. To transform data with 0.0 values into a more normal distribution, the Yeo–Johnson power transformation was used [38]. Transformations were performed on the whole group of participants with no sex separation.

In all clustering analyses described below the same procedure was conducted. As the first step of the procedure, scaling the variables was performed. Normalization in the range (0,1) was conducted. Secondly, Euclidean distances were calculated. Thirdly, clustering participants and/or variables using Ward’s method as a linkage method was performed. Distances between variables or participants were characterized as mean and standard deviation, min and max with range values. The Mantel test was used to assess correlations between two distance matrices with the calculation of the *p*-value. For single dendrograms, cophenetic correlations (c) and Baker’s Gamma (BG) were calculated. In addition, the Fowlkes–Mallows Index (FMI) was calculated to assess differences in vectors between two dendrograms [39]. To assess similarities and congruence between both patterns (PAPs and DPs), tanglegrams were drawn. The untangle method was used to rotate branches of the trees in dendrograms for better visual interpretation [40]. Two-way cluster analysis was used to examine PA and dietary patterns parallelly to identify subgroups of participants according to the differences in such patterns. Graphical results included plots called heatmaps with dendrograms of the variables and objects within the same graph. The following additional R packages were used: dendextend [41], phytools [42], plyr [43], ape [44], viridis [30], BiocManager [45], and ade4 [46].

The clustering procedure k-mean was employed for partitioning a dataset into a set of k groups (i.e., clusters). Final decision was taken based on two-way clustering and tanglegrams from the first step of analysis and k-means results’ graphical presentation with support of the methods for determining the optimal clusters: elbow method and silhouette method.

One-way ANOVA was used to assess differences in BMI, BFP, and FMI between the identified groups, separately in each sex. When significant differences were observed (significant F-ratio), a detailed comparison of post hoc tests (Tukey’s HSD test) was used to determine pairwise differences.

The significance level for all statistical tests and procedures was set at an α-value equal to 0.05. All calculations (except the above-mentioned ones conducted using RStudio) were carried out using Statistica 13.0 (StatSoft Poland 2018, Cracow, Poland).

## 3. Results

### 3.1. Sample Characteristics

The average age of males was 20.36 (±0.83) years and that of females was 19.99 (±0.68) years. Male’s average body height was 180.39 cm (±6.69) and that of body weight was 79.68 kg (±10.83). Female’s average body height was 167.99 cm (±8.28) and that of body weight was 64.15 kg (±9.92).

Table 1 displays the body mass composition and questionnaires characteristics of the study sample. Only global indices of PA and dietary behaviors are shown, due to a too extensive table. A comparison of PA rates showed few significant sex differences. Males had an average value for active transport walking and domestic/gardening that was significantly lower than that of females (*p* = 0.012, *p* = 0.002, and *p* = 0.027, respectively). Males had average values in domestic/gardening vigorous activity and leisure times vigorous activity that was significantly higher than that of females (*p* = 0.042 and *p* = 0.036, respectively). Interestingly, there were no significant differences in the working/school domain, domestic/gardening domain, and leisure time domain (*p* > 0.005 for all), as well as in the overall MET score (*p* = 0.448). The only difference among domains was active transport and males had significantly lower values (*p* = 0.012).

Regarding the frequency of food consumption, males had more non-healthy dietary habits, consuming fried meals (*p* = 0.005), canned meals (*p* = 0.048), and sweetened beverages (*p* = 0.019) more frequently, resulting in a negative total sum of the frequency of consumption for health food groups and a negative HDI-8 index (*p* = 0.042 and *p* = 0.036, respectively). The only positive factor was a higher number of meals per day (*p* = 0.048).

According to IPAQ scoring protocol [31] defining categorical levels of physical activity, there was no person (neither males nor females) with a low PA category (<600 MET min/week). More participants, 81% males and 76% females, were at a high level of PA (≥3000 MET min/week), and fewer (19% males and 23% females) were at a moderate level of PA. Sex differences in presented proportions were not statistically significant (*p* = 0.579).

Similar comparisons were conducted in relation to the QEB scoring protocol. Two global indices were calculated based on single food groups, the pro-healthy HDI-8 index, and the non-healthy HDI-8 index. Each index was separated into three categories: low (range: 0–33 points), moderate (range: 34–66), and high (range: 67–100). Males and females received similar results: 94% of males and 90% of females were placed in the low category, while only 6% of males and 10% of females had a moderate category of pro-healthy food index. These sex differences in proportion were not statistically significant (*p* = 0.413). On the other side, 100% of males and 98% of females had a low category on the non-healthy food index. Small sex differences were not significant (*p* = 0.301).

### 3.2. Congruence in Patterns of Behavior between Males and Females Analysis

**Figure 2:**
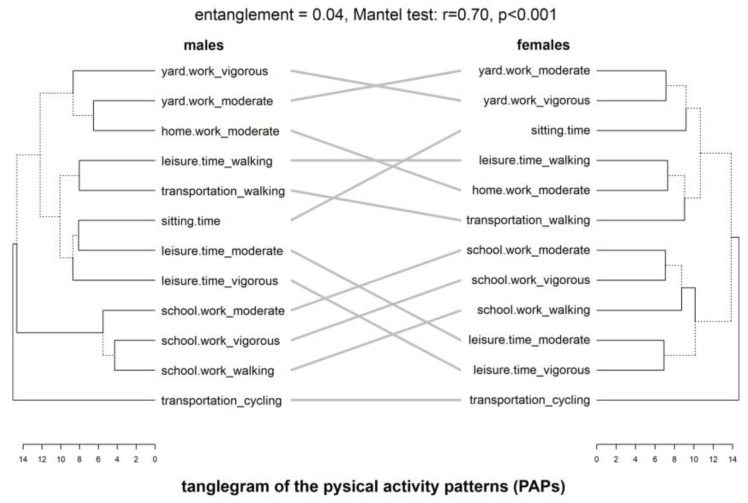
Tanglegrams of the physical activity patterns in males and females. Euclidean distances and Ward’s method of linkage were used. Primary dendrograms were untangled. Bottom, horizontal open bars present distances between items in each cluster. Dashed lines indicate the most differences in males’ and females’ patterns.

**Figure 3:**
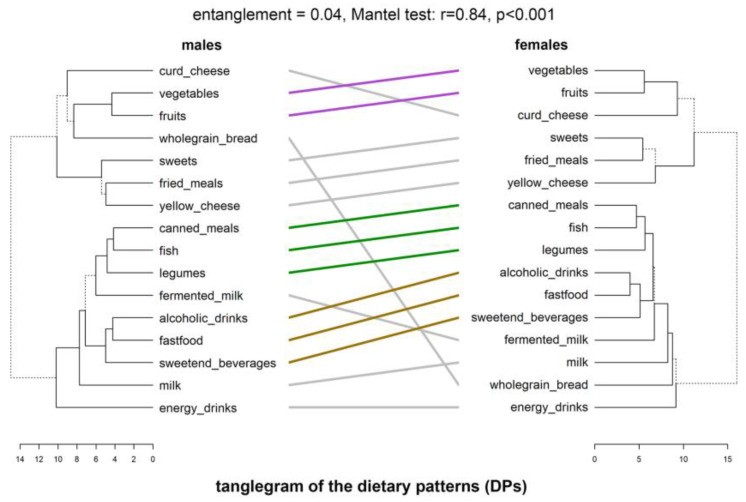
Tanglegrams of the dietary behavior patterns in males and females. Euclidean distances and Ward’s method of linkage were used. Primary dendrograms were untangled. Bottom, horizontal open bars present distances between items in each cluster. Dashed lines indicate the most differences in males’ and females’ patterns. Colored connecting lines highlight two sub-trees which are present in both dendrograms.

Congruence, expressing similarity between the same kind of behaviors (PA and dietary), was analyzed twofold: (1) started with a visual comparison of two dendrograms (males vs. females) related to the PAPs and separately to the DPs, by facing them one in front of the other and connecting the same labels and (2) supplemented with cophenetic statistical analysis. Graphical presentation were tanglegrams shown in Figure 2 and Figure 3. The males’ dendrogram of PAPs or DPs was linked with the help of cophenetic relations with the females’ dendrogram. Similar subtrees were connected by lines of the same color. It showed similarities in patterns. Branches leading to distinct subtrees were marked with dashed lines. It showed dissimilarities. The applied untangle method simplified the connections between both dendrograms, decreasing entanglement. Tanglegrams were much clearer than the primary computed before the untangle procedure.

#### 3.2.1. Physical Activity Patterns

The tanglegram of the two dendrograms generated on the base of the PA items between males and females is shown in Figure 2. The primary entanglement value of 0.45 was reduced to 0.04, what could be considered as a high similarity between patterns. From Figure 1, it is clear that in both sexes three clusters were separated. The first similarity between both sexes was a lone existing branch related to transportation by cycling corresponding in both dendrograms. In males, the second cluster was small and included all three school/work activities. These PA items were linked with the same items in females (also in the second cluster), but, in the opposite, connected with leisure time activities. It showed that females active in their school or work duties spent their leisure time on vigorous or moderate activities. The third cluster of males was divided into three subclades. The first contained: vigorous and moderate activities in leisure time with inactivity during the sitting time. The second subclade contained walking activities during transportation or in leisure time. This part of the patterns was similar to females’ first subclade in the third cluster. The third subclade in males included vigorous and moderate activity outside home with home moderate working. This part of the patterns was also similar to females’ patterns in the second subclade of the third cluster.

Similarities in PAPs were also confirmed with cophenetic statistical analysis. The average male’s PAPs distance was 9.67 ± 1.87 (mean and sd), ranging 9.40 from 4.28 to 13.68 (min and max, respectively). In females, it was 9.70 ± 1.71, ranging 7.35 from 6.92 to 14.27. These basic statistics showed great similarities in distances between males and females. The Mantel test conducted to assess the statistical significance of the correlation between matrices of distances between both sexes showed a strong and highly significant relationship (r = 0.70, *p* < 0.001). These remarks for distances were also confirmed with the Fowlkes–Mallows Index (FMI), calculated based on vectors of clustering groups. FMI showed good and statistically significant similarity of the dendrograms (0.68, *p* = 0.005).

#### 3.2.2. Dietary Behaviors Patterns

The tanglegram between the dendrogram obtained from PA items and the dendrogram obtained from dietary items (food frequency consumption) is shown in Figure 3. The primary entanglement value of 0.79 was reduced to 0.04. Hence, the patterns were presented more clearly. The comparison of the 16th dietary items between males and females showed great similarity between the patterns; however, distinct disparities were also observed. In both sexes, two main clusters were revealed which contained mostly the same elements. The most similar parts of the patterns were marked with colored lines (purple, green, and gold), which linked the same items included in the same subclades in analogous clusters. The first similarity between both sexes, same as for PAPs, was a lone existing branch related to energy drinks consumption in the first cluster. Both sexes, males and females, consumed energy drinks independently of other products. It is noteworthy that non-healthy behaviors were reinforced because of the concatenated triad (colored in gold): fast-food, sweetened beverages, and alcoholic drinks. The sex-specific difference was different in conjunction with other, healthy food groups. In males this included: dairy (milk and fermented milk drinks), legumes, and fish, but also canned meals. In females, there was a similar pattern in relation to the last three elements (marked in green), but also with milk and wholegrain bread. The second cluster in both males and females contained the second non-healthy triad: sweets, fried meals, and yellow cheese. This triad coexisted, however, (in both sexes) with a healthy diad of fruits and vegetables, linked in addition to curd cheese and wholegrain bread (only in males).

The average male’s DPs distance was 7.5 ± 1.61 (mean and sd), ranging 6.81 from 4.15 to 10.96 (min and max, respectively). In females, it was 7.95 ± 1.69, ranging 8.15 from 4.0 to 12.15. Shorter distances compared with PAPs suggested stronger links between dietary behaviors. A Mantel test showed stronger than for PAPs and also a highly significant relationship (r = 0.84, *p* < 0.001). Great similarities were confirmed also with FMI (0.68, *p* < 0.001).

### 3.3. Congruence between PAPs and DPs in Males and Females Analysis

**Figure 4:**
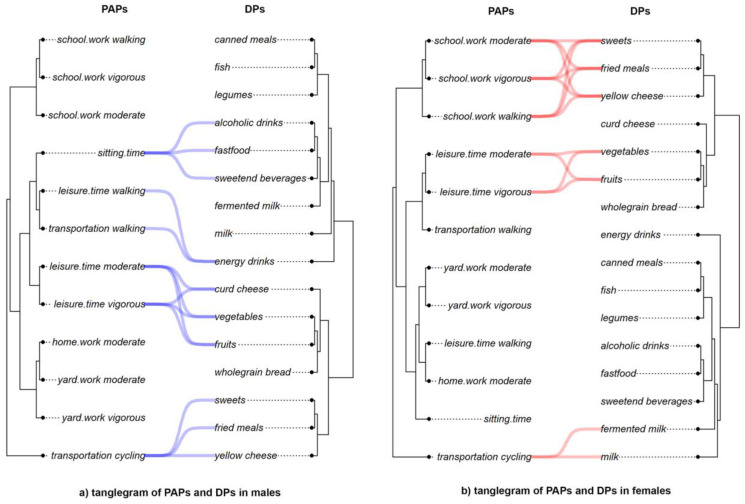
(**a**,**b**) Tanglegrams of the PAPs connected to DPs in males (**a**) and females (**b**). Euclidean distances and Ward’s method of linkage were used. Original dendrograms were untangled for better visualization. Colored lines connect the most closely linked (shortest distances) PAPs items with DPs food groups.

Tanglegrams of the relationship between PAPs and DPs in each sex are shown in Figure 4a,b. The untangle function was used to better visualize the connections. A comparison of the 12 PA items and 16 dietary food frequency consumption showed the strongest relations (closest distances), which were presented with colored lines. Analysis of the tanglegram for males suggested four patterns of congruence between PAPs and DPs (Figure 4a). The first one was a relationship between using a bicycle for transportation with eating sweets, fried meals, and yellow cheese. The second one identified connections between leisure time vigorous and leisure time moderate activities and the eating of healthy foods: fruits, vegetables, and curd cheese. In the third pattern, males who walked a lot (leisure time walking and transportation by walking) often drank energetic drinks. The last one was a pattern connecting average sitting time and very unhealthy dietary behaviors, such as consuming alcoholic drinks and sweetened, carbonated beverages, and eating fast food meals. The congruence between PAPs and DPs looked different in females (Figure 4b). There were three patterns of congruence. Females who were using bicycles for transportation usually consumed milk and fermented milk drinks. The second was similar to males and identified a close connection between leisure time vigorous and leisure time moderate activities and eating healthy foods such as fruits and vegetables (besides curd cheese). The last one was a pattern of connections between all three school/work activities and eating sweets, fried meals, and yellow cheese.

### 3.4. Structure of the Individuals in Relation to Behaviors Patterns

#### 3.4.1. Physical Activity

**Figure 5:**
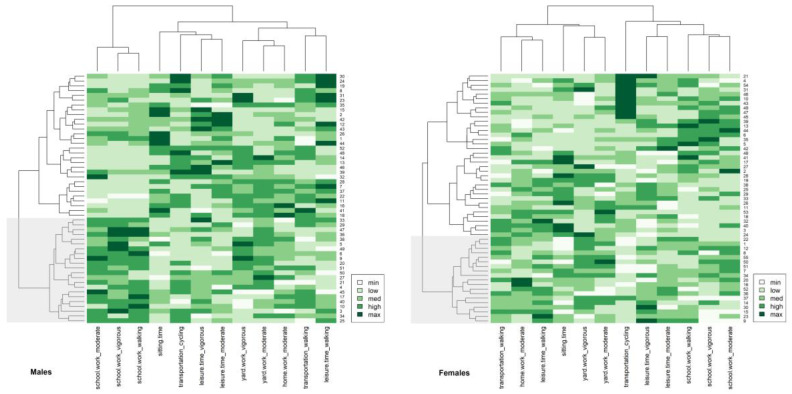
Two-way cluster analysis dendrograms (Ward’s method) with heatmaps (Euclidean distances) based on physical activity items (derived from IPAQ questionnaire). The numbers on the right side of heatmap represent participants, the short phrases at the bottom represent PA items. Legend shows the strength of linkage—darker color represents a shorter pairwise distance (between participants and PA items).

The patterns of PA in the male and female groups were assessed using 12 IPAQ items (including average sitting time). After the transformation of the data, mean values were calculated, scaled (standardized), and included in the two-way cluster analysis. Two-way clustering of PA items and the participant’s dendrograms together with heatmaps of the distances for both sexes are presented in Figure 5.

Sex differences in PAPs were revealed. In males, three main PAPs and two main patterns in females were identified and are shown in Figure 2. In both sexes, however, the main patterns were separated into four detailed patterns. In males, the first cluster—School and Work Activity—was described by time spent on various levels of activity at school or work: vigorous, moderate, and walking. The second cluster grouped average sitting joined to active transportation with cycling and leisure time vigorous with leisure time moderate intensity. It could be called Active Recreation. The third cluster was separated into two clades. The first presented is the Domestic and Gardening Activity pattern. This included all domestic and gardening activities (vigorous and moderate outside and moderate inside). The second included walking during active transportation and leisure time walking, so was called Walking Activity. In females, the first cluster was separated into two clades. The first represented a pattern called Mediocre activity. It was described as walking during active transportation, leisure time walking, and moderate domestic and gardening inside the house. The second clade, Domestic Activity, included exhausting domestic and gardening activities (vigorous and outside the house) linked to average sitting. The second cluster was also separated into two clades. The first presented an Active Recreation pattern and was described by time spent with two levels of activities during leisure time—vigorous and moderate—and time spent with active transportation with cycling. The second clade represented School and Work Activity and was described by all three work/school activities (vigorous, moderate, and walking).

Two-way clustering analysis examined the convergence of the PAPs with the structure of the participants. In both sexes, males and females, two primary clusters were identified. The first cluster contained 22 male individuals and 19 female individuals (the second cluster contained 30 and 36 individuals, respectively). However, the second cluster was separated into two groups of participants. Therefore, generally, three groups of participants of each sex were indicated. In males, the average PA distance between males was 4.79 ± 1.04 with a range from 2.04 to 8.36. The cophenetic correlation between cophenetic and Euclidean distances was 0.50, indicating that the real similarities between participants in terms of PA items were well-mapped in the dendrogram. The first group of males (colored gray in Figure 2) was closely associated with School and Work Activity. The second group was associated with Active Recreation and Domestic and Gardening patterns, while the third was linked to Active Recreation and Walking Activity. In females, the average PA distance between females was 4.77 ± 1.13 with a range from 1.52 to 8.59. The cophenetic correlation was smaller than that in males (0.44). The first group of females (colored gray in Figure 2) was associated with Mediocre activity and Domestic Activity, the second group mostly with Domestic Activity, and the third with active transportation with cycling and School and Work Activity patterns.

#### 3.4.2. Dietary Behaviors

**Figure 6:**
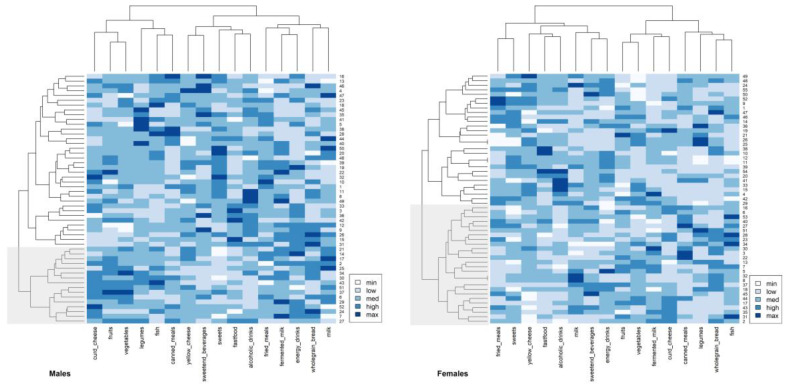
Two-way cluster analysis dendrograms (Ward’s method) with heatmaps (Euclidean distances) based on the frequency of consumption of food groups (derived from QEB questionnaire). The numbers on the right side of the heatmap represent participants, short phrases at the bottom represent food groups. The legend shows the strength of linkage—the darker color represents the shorter pairwise distance (between participants and PA items).

Figure 6 presents two-way clustering of the 16 dietary food groups (eight pro-healthy and eight non-healthy) between participants. Heatmaps represented the distances (darker color—shorter distances).

Five DPs were found in males. The two positive patterns were Curd and Plants and Bread and Dairy. The first was described by the frequent consumption of curd cheese, fruits, and vegetables, while the second was by consuming wholegrain bread and milk. One negative pattern was revealed—Fast-food and Drinks. It was described by the consumption of fast food meals, alcoholic drinks, and sweetened, carbonated beverages linked with yellow cheese and sweets. Two mixed patterns were also found. Canned and Legumes contained canned food, peas, beans dishes, and fish dishes. The last one was Dairy and Fried-Energetic described by the consumption of fermented milk drinks, fried meals, and energy drinks. In females, in addition to the five patterns, differences were found. There was one clear positive pattern called Dairy and Plant. It was described by the consumption of fermented milk drinks, curd cheese, fruits, and vegetables. However, two clear negative patterns were identified. The first was Fried and Sweets, described as eating fried meals and the frequent consumption of sweets. The second was Fast-food and Drinks, containing fast food meals and alcoholic drinks related to yellow cheese. Two mixed patterns were also identified. The first one, Milk and Energetic, contained milk, energy drinks, and sweetened, carbonated beverages. The second one, called ‘Canned and Legumes’ was described by the consumption of peas and bean dishes, canned food, fish meals, and wholegrain bread.

The study of participant structures identified three groups of students associated with the revealed DPs. In males, the average frequency of food consumption distance in males was higher than what was calculated for PA items and was 5.54 ± 1.147, ranging from 2.01 to 10.28. This suggested a looser linkage between the participants than that of PA. The cophenetic correlation between cophenetic and Euclidean distances was 0.367, which indicated that real similarities between participants in terms of food consumption frequency were worse mapped in a dendrogram than that in cases of PA. The first group (marked in gray) was an independent cluster. It was connected mainly to Curd and Plants. The second cluster gathered another two groups of participants. Group number two was mostly related to Bread and Dairy and Dairy and Fried-Energetic patterns. The third group was linked mostly with Fast-food and Drinks. In females, the average food frequency consumption distance in females was 5.55 ± 1.08, ranging from 1.00 to 8.46. The cophenetic correlation was 0.40. The first cluster (colored in gray) was linked partially to Dairy and Plant, Milk and Energetic, and Canned and Legumes. The second cluster was separated into two groups of females. The first—as the second next group, was linked mainly to Fast-food and Drinks, while the third next group (second in this cluster) was associated mainly with Fried and Sweets.

### 3.5. Associations between Physical Activity, Dietary Behaviors, and Body Composition Analysis

The last part of this work was to assess the associations between PA and dietary behaviors and body mass composition. The strategy was to separate males and females into three groups (based on the silhouette index) using the *k-means* clustering method. The calculations were conducted twice and based on (1) patterns revealed in an earlier paragraph and (2) the total PA results (overall MET min/week), total healthy HDI-8 index, and total unhealthy HDI-8 index. However, validation of the self-reported BFP data was conducted.

#### Validation of the Self-Reported Percentage of Body Fat Data

**Figure 7:**
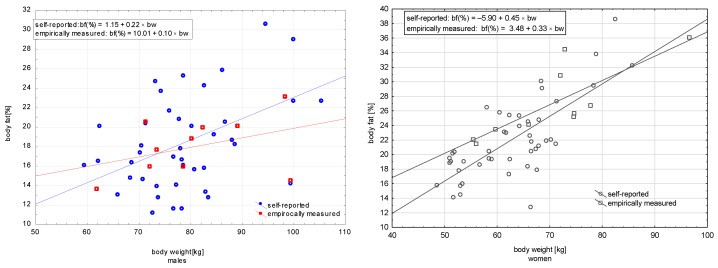
Body fat (%), self-reported and empirically measured, regressed on body weight in men and women, respectively (bf (%)—the percentage of body fat, bw—body weight).

The reliability of the self-reported percentage of body fat was positively validated with a regression procedure, which is graphically presented in Figure 7. In both sexes, there were no significant shifts between the regression lines, confirming insignificant differences between constants in both groups (self-reported and objectively measured data, boys: *p* = 0.599 and girls: *p* = 0.185). In addition, lines drawn based on the self-reported and measured data were parallel, which confirmed statistically insignificant interactions between regression lines (boys: *p* = 0.139 and girls: *p* = 0.202). Therefore, the sample evidence is strong enough to accept the hypothesis that the group differences (self-reported and objectively measured in boys and girls) equal zero (i.e., no differences), while insignificant slopes confirmed that the way of registering data did not affect the relationship between body weight and its fat component.

Confirmed consistency between BFP and body weight, comparability between self-reported and empirically measured data, and the reliability of self-reporting methods allowed us to accept the reliability of the data and perform the last analysis. In searching for associations between physical activity and dietary behaviors with body mass composition (BMI, BFP, and FMI), comparisons between separated subgroups were made several times. Firstly, full sets of PAPs and DPs were used; secondly, only the most related items of the PAPs and DPs (retrieved from tanglegrams) were used. Next, only PAPs items and, separately, DPs items were tested. In the end, total indices of PA and dietary (positive and negative HDI-8 indices) were tested.

Most of the approaches failed to find associations between related PAPs and DPs and body mass index, percentage of body fat, and fat mass index. There were no significant differences either in males or in females.

Only an approach using both dietary positive and negative HDI-8 indices (without PA), showed effects. The effect was partial, because it did not concern males. In males, there were no significant differences, whilst significant differences were found in females (BMI: F = 3.19, *p* = 0.049; BFP: F = 3.87, *p* = 0.027; FMI: F = 3.83, *p* = 0.028). The first cluster (C_1_) included 13 female individuals (BMI: 24.03 ± 2.78; BFP: 25.33 ± 6.47; FMI: 6.22 ± 2.29), the second (C_2_)—21 (22.88 ± 2.77; 23.27 ± 6.09; and 5.45 ± 2.03, respectively) and the third (C_3_)—21 (21.66 ± 2.56; 20.11 ± 4.16; and 4.43 ± 1.40, respectively). Detailed comparisons revealed that females from C_1_ significantly differed only from females included in C_3_ (BMI: *p* = 0.016; BFP: *p* = 0.010; FMI: *p* = 0.009). Females grouped in C_1_ had lowest level of PA (5801 ± 2227 MET min/week), medium healthy HDI-8 index (22.25 ± 5.48), and highest unhealthy HDI-8 (22.17 ± 5.49) compared with C_2_ (7466 ± 3550 MET min/week; 27.44 ± 6.74; and 9.07 ± 3.09, respectively) and C_3_ (6160 ± 3411 MET min/week; 10.64 ± 4.59; and 11.974.62, respectively). The differences in physical activity were not statistically significant (F = 1.34, *p* = 0.270), but were significant in healthy HDI-8 index (F = 47.16, *p* < 0.001) and unhealthy HDI-8 index (F = 38.54, *p* < 0.001). Detailed comparisons of the healthy HDI-8 index showed significant differences between C_1_–C_3_ (*p* < 0.001) and C_2_–C_3_ (*p* < 0.001), but not C_1_–C_2_ (*p* = 0.062), whilst in unhealthy HDI-8 in C_1_–C_2_ (*p* < 0.001), C_1_–C_3_ (*p* < 0.001), but not between C_2_–C_3_ (*p* < 0.086)

## 4. Discussion

In this study, a comparison between both sexes showed average higher intensity of the PA vigorous domestic/gardening activity and vigorous leisure time activity in males than females. However, females were more active in the transportation domain (mainly in active transport walking). There were no sex differences in overall MET. Regarding the frequency of food consumption, males usually consumed usually fried meals, canned meals, and sweetened, carbonated beverages more frequently, which resulted in the worse total sum of negative items and was reflected in the final negative HDI-8 index score. The only positive aspect was a higher number of meals per day. A comparison of the patterns showed great similarities between the PAPs and DPs between males and females. In PAPs, this included: transportation cycling and school/work activities, although they were related to other PA items in both sexes and with different distances (in boys more closely related). The main differences were related to average sitting, leisure time walking, and domestic and gardening activities. DPs were more similar than PAPs in both sexes. Two triads of non-healthy behaviors were revealed similarly in males and females: first—fast-food, sweetened, carbonated beverages, and alcoholic drinks and second—sweets, fried meals, and yellow cheese. However, a diad of healthy behaviors was also observed: fruits and vegetables. In both sexes, consumption of energy drinks was independent from consumption of other products. Dissimilarities mainly concerned the different detailed structure of subtrees in the same cluster. Congruence between PAPs and DPs in a specific scope, as well as sex differences in such a congruence, was observed. In males from among the four patterns, two were noteworthy. Healthy PA behaviors in leisure time were associated with healthy eating (fruits, vegetables, and curd cheese), and unhealthy PA behaviors related to average sitting were connected to drinking alcoholic and sweetened, carbonated drinks, and eating fast food. In females, more mixed patterns were observed. From among four patterns, two were also noteworthy: healthy leisure time PA behaviors (vigorous and moderate activity) were connected to eating fruits and vegetables; however, activities at school/work were related to eating sweets, fried meals, and yellow cheese. Linking patterns in lifestyle behaviors with the detailed structure of males and females, four PA patterns in males (‘*School and Work Activity*’, ‘*Active Recreation*’, ‘*Domestic and Gardening Activity*’, and ‘*Walking Activity*’) and four PA patterns in females (‘*Mediocre activity*’, ‘*Domestic Activity*’, ‘*Active Recreation*’, and ‘*School and Work Activity*’) were revealed. In the case of dietary patterns, there were five patterns in the male group. Two positives were: ‘*Curd and Plants*’ and ‘*Bread and Dairy*’. One negative was *Fast-food and Drinks*. Two mixed were: ‘*Canned and Legumes*’ and ‘*Dairy and Fried-Energetic*’. Similarly in females, five dietary patterns were also found; however, another was constructed. There was one positive: ‘*Dairy and Plant*’ and two negative: ‘*Fried and Sweets*’ and *Fast-food and Drink*’. Additionally, two mixed patterns were found: ‘*Milk and Energetic*’ and ‘*Canned and Legumes*’. Lifestyle was reflected in body mass index and fat mass, but only in females. However, the present study failed to find associations between patterns of congruence between PAPs and DPs and BMI, BFP, and FMI. General dietary behaviors (related to total healthy HDI-8 index and total unhealthy HDI-8 index) were more important and affected the level of body mass composition, whilst total PA (MET min/week) did not.

The place of study is recognized and related to health behaviors. Recent works showed multifactorial effects of the study on the level of PA, sedentary behaviors, and dietary habits [47]. In the presented study, moderate and high levels of PA in the students were observed, but were not significantly higher in the female group (overall MET min/week). MET values for both sexes were twice as high as those observed by Zuzda et al. [48] and Pastuszak et al. [49] in Polish university students. Taking into account the type of activity (vigorous, moderate, and walking), students in the presented study had lower levels of vigorous and moderate types of activity than did students studying physical education as examined by Maciaszek et al. [50] in the physical education study field. Simultaneously, the authors presented results from the Czech Republic, Germany, and the Netherlands. Analysis showed the highest level of PA in Czech Republic students (males and females), then the Netherlands, and finally the German students. Students from all three countries had higher levels of PA than the Polish students examined by Maciaszek et al. and in comparison to the students presented in this article. Czech students were recognized as being the most active students from European countries [51,52]. Sedentary behaviors in the presented study expressed as a total sitting component (min per week) were worse than other Polish student populations [48] and Scandinavian students. Some studies showed a significant difference between male and female students regarding PA but not regarding sedentary behaviors [53]. The presented study is in partial agreement with Edelmann et al., suggesting that there are no sex differences in total PA and total sitting behavior. However, this is contrary to Maciaszek et al. [50], who observed a significantly higher level of PA in males than females. The explanation could be that the COVID-19 pandemic limited PA (particularly during leisure time and in relation to vigorous activity) and the post-pandemic recovery could be taking more time. Confirmation of this fact is presented in a study from the same university conducted just before the pandemic (in 2017–2018) [54] and found better results in vigorous activity (2527 MET min/week compared to 1680 MET min/week in males and 1816 MET min/week in females presented our study), but worse results in moderate activity and walking (2017–18 year: moderate—1295 MET, walking—1684 MET vs. present: moderate—2467 MET and 2280 MET (in males and females, respectively).

This study showed similar frequencies of consuming healthy products while expressing sex differences in the frequency of consuming non-healthy products. Males, unlike females, were consuming fried meals, canned meals, and sweetened, carbonated beverages significantly more often; consuming fast foods was very close to reaching significance, resulting in higher values on the non-healthy HDI-8 index. These results are similar to several studies related to adolescents and children [55,56] A number of studies more deeply examined the relationship between non-healthy dietary habits and physical inactivity, particularly screen time [57,58,59]. Screen time inactivity and non-healthy dietary behaviors are concerning factors related to overweight and obesity, particularly in adolescents [60,61]. In this study, males who presented with non-healthy dietary behaviors also had relatively high levels of time spent in sitting positions (1868 MET min/week); however, this is not the worst result compared to other Polish and foreign cohorts [62]. These authors observed an average sitting time in students exceeding 2500 MET min/week. The connections between PA and dietary behaviors as reflected in body mass composition, especially in excess body fat, lead to the conclusion that a multidimensional approach is needed in dealing with obesity in adolescents.

The cophylogenetic approach of facing two dendrograms and using adequate statistics allowed objectifying sex differences in PAPs and DPs. Results confirmed strong similarities with distinctive dissimilarities related in PA, mostly to active transportation by walking, walking activity in leisure time, average sitting, and moderate domestic and gardening activity. That kind of sex difference might suggest the different function of the sitting time in males and females. These results are contrary to results for Polish adults (21–64 years of age) before the pandemic (2018), where men and women have similar sedentary behaviors [63]. They spent a similar amount of time reading books and newspapers, using computers, and watching TV. The explanation could be more age variation in that study with a bias towards younger people. Other Polish studies showed that a number of hours spent in a sitting position, for both sexes, increases as age progresses [64]. Own results might suggest that males who were very active in leisure time needed a lot of time for rest and they could prefer passive resting in a sitting position, whilst in females sitting time could be a means of relaxation after strenuous work in the house and around the house. Respectively, in dietary behaviors, the main sex differences were related to alcoholic drinks, sweetened, carbonated beverages, fast-food meals, and fermented milk drinks, of which consumption was higher in men. These results are partially in agreement with other Polish studies on young adults [65]. The dietary choices of women, more often than those of men, corresponded to the principles of healthy nutrition. It was related to a greater number of meals consumed during the day, more frequent consumption of fruits and vegetables, and the selection of products with lower energy value. In own studies common, a positive pattern for both sexes was a similar consumption of fruits, vegetables, and curd cheese. Confirmation of the strong but not perfect similarities were cophenetic statistics showing 0.68 and 0.83 correlation coefficients between male and female patterns in PA and dietary behaviors, respectively.

This study revealed that in young males and females starting college, some specific PAPs were associated with some specific DPs. The sex differences were also observed. In males, four patterns of congruence between PAPs and DPs were identified and could be described as positive, mixed, and negative patterns. The positive pattern was the association between vigorous and moderate PA during leisure time and the frequency of consumption of fruits, vegetables, and curd cheese. Such a type of relationship did not confirm the patterns identified in Polish adults before the pandemic [63]. The authors revealed that more frequent consumption of fruits and vegetables was conducive to sedentary behaviors (watching TV and reading books and newspapers). Perhaps the pandemic changed the behaviors of young adults. This statement is supported by results received by other Polish authors who observed numerous changes to the food chain in Poland, especially in terms of food purchase and consumption [66,67]. The research should be, however, continuing. The unhealthy pattern was the association of sitting time and alcoholic drinks, sweetened, carbonated beverages, and fast food consumption. The two mixed patterns showed associations between walking and the consumption of sweets, fried meals, and drinking energy drinks. In females, four patterns were also revealed. The positive congruence patterns of activity during leisure time and transportation by walking were connected to fruit and vegetable consumption. Females who were engaged in work/school activities preferred the non-healthy consumption of sweets, fried meals, and yellow cheese. A third pattern suggested that sitting time was connected to eating wholegrain bread. The fourth pattern joined transportation by bicycle with the consumption of milk and fermented milk drinks. The structure of the associations in healthy congruence patterns in males and females was in agreement with those commonly observed. Physical activity during leisure time correlated with the consumption of fruits and vegetables. The associations between an active lifestyle and a healthy diet have been well documented in Polish and foreign populations [2,68]. Authors have shown sex differences, but have suggested a less clear association in females. However, in some studies, students active at school were more likely to consume fruits and vegetables than students who were not active [2,69,70]. The results of this work suggest a relationship between school/work behaviors and the unhealthy eating of sweets, fried meals, and yellow cheese. However, special attention should be paid to the combined unhealthy habits observed mostly in males. This concerned the average sitting time and its association with alcoholic drinks, sweetened, carbonated beverages, and fast food consumption. This observation has also been confirmed by other authors identifying connections between healthy and unhealthy items to create clustering patterns of PA and diets observed when using different methodologies [71,72]. This kind of pattern could be dangerous from a health point of view leading to obesity and other health consequences. Findings from these studies suggest that obesogenic cluster patterns were complex with a mixed low PA/sedentary behavior related to unhealthy eating. A number of studies presented in a recent review [73] have defined a close relationship between unhealthy diet, low PA, and sedentary behavior among children and adolescents leading to unhealthy behaviors and the development of overweight and obesity [74,75].

Detailed analysis of PAPs and DPs in relation to participants structure showed four main PA patterns in males and four main PA patterns in females were revealed. In the case of dietary food consumption frequency, five patterns in males and five patterns in females were revealed. This is generally consistent with a number of clusters most often observed by Polish and foreign authors in studies of adolescents [2,76]. The number of clusters usually ranged from two to seven [77,78]. Many studies identified different patterns for males and females, although sex differences among adolescents were not clearly crystalized [73,79]. In spite of this, the literature on physical activity presented evidence that gender influenced participation in PA. This is related, however, predominantly to quantitative rather than qualitative results [80,81] The explanation is that males and females like different kinds of physical effort and prefer different forms of physical expression, with the conclusion that there is a need to examine physical activity and sedentary behavior separately in boys and girls [79]. In this work, different patterns in PA and dietary patterns in males and females were observed, which is in agreement with the above-mentioned observations. Sedentary behaviors are defined very often as independent patterns in some studies [78,82]. Results of this work are partially in agreement because, as in males as in females, average sitting time created a lone cluster, not fully independent but related to ‘*Walking Activity*’ in males and ‘*Domestic Activity*’ in females. This is interesting and might suggest that academic adolescents who study in PE- and sport-oriented fields at university do not prefer inactive sedentary behavior, and those who spend much more time in a sitting position during the day compensate and equalize this time later with physical activity. This observation is in line with other authors suggesting that PA can coexist with high levels of sedentary behavior or vice versa [81,83]. This kind of pattern partially confirmed results received in Polish males before the pandemic [68]. In agreement with other studies, there is an observed ‘*School and Work Activity*’ pattern that was clearly structured in both sexes [2,70]. In the case of females, the connection between activity in school and work and vigorous and moderate activity in leisure time, as well as transport by cycling, could be related to some psychological traits, e.g., high level of ambition, solidity, or ambition to perform work duties or school classes better. Relationships between different psychological characteristics and rationalization of the diet were observed in athletes training individual sport disciplines [84].

Dietary patterns were usually found to cluster clearly in healthy and unhealthy ways, creating separate and clear patterns [2,75,85]. However, in some studies most adolescents fell into the mixed category, showing several healthy dietary behaviors concomitant with one or more non-healthy behaviors [73]. This is the situation dealt with in this article. Therefore, as in males and in females, patterns such as ‘*Canned and Legumes*’ or ‘*Milk and Energetic*’ appeared, where, no doubt, unhealthy canned meat and fish dishes were linked to peas and beans and milk meals. Some studies identified clear healthy and unhealthy patterns existing together with the mixed category [83]. In this work, clear pro-healthy dietary behaviors were ‘*Curd and Plants*’ and ‘*Bread and Dairy*’ in males, gathering curd cheese, fruits and vegetables, wholegrain bread, and fermented milk drinks; one in females was *‘Dairy and Plant’*, gathering fermented milk, curd cheese, fruits, and vegetables. This pattern was similar to that revealed by Wądołowska et al. [2] in female adolescents called *‘Fruits and vegetables’*. The clear unhealthy behavior was *Fast-food and Drinks* in males and *‘Fried and Sweets*’ with *Fast-food and drinks* in females, which contained fast-food meals, sweetened, carbonated beverages, and alcoholic drinks. This cluster was very similar to that observed in the Wądołowska et al. *‘Fast food and Sweets’* pattern.

Analyses of whether body mass composition reflected lifestyle patterns only partially confirmed an association between PA and dietary behaviors and BMI, PBF, and FMI. The revealed patterns of congruence between PAPs and DPs presented above did not affect BMI or FMI in females or males. Generally, dietary behaviors (related to the total healthy HDI-8 index and total unhealthy HDI-8 index) were more important in affecting body mass composition, whereas total PA (MET min/week) did not. These results are contrary to studies suggesting that PA was inversely associated with PBF [1,3,4]. However, in contrast, other studies did not show similar associations between job-related PA [80,81], as did the current study. On the other hand, these results have confirmed the eminent role of dietary behaviors, particularly in females.

A major strength of this study was the novel (in the field of PA and dietary behavior association assessment) approach using cophylogenetic variants of clustering. This method of assessing the connections between PA and dietary behaviors allowed for a detailed evaluation of the congruence of patterns between PAPs and DPs in youths starting their academic education. Clear visualization using tanglegrams allowed for an easy way to assess sex differences in the observed patterns. To the best of the author’s knowledge, this is the first study to date with such an exploratory strategy in PA and dietary relationships.

One of the limitations of the present study was the cross-sectional design, which prohibits causal inference, but enables the evaluation of associations. Studies employing interventions and causal relationships should be implemented in the future. Another limitation was the small number of participants. Although the power calculations positively confirmed the validity of the analyses, the numbers of males and females made more detailed comparisons difficult. Another limitation was that the objective measurement of BMI and FMI was not universally applied. Self-reported data were positively verified, but objective measurements might give more accurate results. Narrow groups do not allow for the development of inferences but give new insight into the patterns and their effects on physically active (peers who do not study at physical education and sport-oriented universities) students. The last limitation is the indirect assessment of PA. A direct, more accurate measurement with accelerometers would be more suitable..

## 5. Conclusions

A novel (in the field of PAPs and DPs studies) cophenetic exploratory approach proved useful in the study of sex differences in youths starting college. This study demonstrated that students learning in the physical education and sport science field of study achieved the minimal level of PA recommended by experts from the WHO. Identification of the reasons for a lower level of PA in comparison to other populations, as well as poor dietary behaviors, is, however, needed. This might help to eliminate the limitations and improve healthy lifestyles. Transformation of the type of activity from moderate (and walking) to vigorous is also required. This study confirmed that academic adolescents studying at a PE and sport-oriented university presented non-uniform and more complex patterns of physical activity and food consumption frequency than it might seem. Mixed patterns containing healthy and unhealthy PA or dietary food groups prompt the need for targeted tailoring of an intervention program that improves healthy habits. The presence of two non-healthy triads; fast food, sweetened, carbonated beverages, and alcoholic drinks in the first; and sweets, fried meals, and yellow cheese in the second; in males and females was alarming. Even the presence of the healthy diad vegetables with fruits (coexisted in addition with curd-cheese consumption by females, as well as with wholegrain bread and curd-cheese in males) did not mitigate this observation. Linking lifestyle behaviors with the detailed structure of the males and females having specific habits revealed multiple lifestyle patterns. The different functions of sitting time (as an inactivity item) in both sexes were revealed. In males, it might be a way to rest after intensive leisure time activity, while in females it could be related to relaxing after exhausting domestic and gardening work. Body mass composition mirrored lifestyle, particularly in girls. However, a total combined effect of PA and dietary behaviors were observed. The role of dietary behaviors was more important than physical fitness.

## Figures and Tables

**Table 1:** Characteristics of body mass index, fat mass (in kg and %), fat mass index, physical activity rates, and frequency of food consumption in males and females. *p*-values derived from independent t-test and Mann–Whitney’s U test (for frequency of food consumption).

		Males		Females	
	Mean 95%CI	Median Interquartile Ratio	Mean 95%CI	Median Interquartile Ratio	*p*
body mass index [kg/m^2^]	24.45 (23.69–25.22)	24.21 (3.52)	22.69 (21.93–23.45)	22.12 (3.81)	**<0.001**
percentage of body fat [%]	18.45 (17.13–19.77)	17.70 (5.94)	22.55 (20.98–24.12)	21.79 (6.58)	**<0.001**
body fat mass [kg]	14.92 (13.45–16.39)	13.48 (6.05)	14.84 (13.26–16.43)	14.20 (6.45)	**0.001**
fat mass index [kg/m^2^]	4.59 (4.15–5.02)	4.15 (1.94)	5.24 (4.71–5.78)	4.86 (2.40)	**<0.001**
work/school domain [min/week]	1617.81 (1182.25–2053.37)	933.25 (2170.00)	2316.96 (1868.40–2765.53)	1973.00 (2486)	0.432
transport domain [min/week]	1490.97 (1187.55–1794.40)	1353.00 (1301.25)	1606.94 (1181.89–2031.98)	1188.00 (1578.00)	**0.027**
domestic/yard domain [min/week]	1056.78 (799.57–1313.99)	840.00 (1413.75)	937.32 (703.27–1171.36)	620.00 (690.00)	0.660
leisure time domain [min/week]	1926.31 (1617.34–2235.28)	1857.50 (1448.25)	1713.04 (1473.87–1952.21)	1662.00 (1251.00)	0.491
total vigorous [min/week]	1680.77 (1338.48–2023.06)	1440.00 (1240)	1816.87 (1476.82–2156.93)	1600.00 (1440.00)	0.273
total moderate [min/week]	2467.16 (2059.16–2875.16)	2270.00 (2022.50)	2279.50 (1874.24–2684.76)	1905.00 (1600.00)	0.573
total walking [min/week]	1943.94 (1621.95–2265.92)	1724.25 (1410.75)	2477.88 (1981.91–2973.86)	1848.00 (2614.20)	0.514
total sitting [min/week]	1868.27 (1703.39–2033.15)	1700.00 (910.00)	1960.00 (1784.33–2135.67)	1920.00 (1070.00)	0.076
overall MET [min/week]	6091.87 (5395.63–6788.10)	5714.25 (3317.50)	6574.26 (5694.10–7454.42)	5810.00 (4155.00)	0.448
positive HDI-8 index	22.38 (19.67–25.08)	21.44 (14.19)	19.80 (17.26–22.34)	19.88 (14.25)	0.236
negative HDI-8 index	16.04 (13.88–18.20)	14.19 (9.56)	13.28 (11.47–15.08)	11.00 (9.50)	**0.036**

## Data Availability

The data presented in this study are available on request from the author.
